# *Pneumocystis jirovecii* pneumonia following corticosteroid therapy

**DOI:** 10.1590/0037-8682-0553-2022

**Published:** 2023-02-20

**Authors:** Magda Garça, João Domingos, Susana Peres

**Affiliations:** 1 Hospital de Santo Espírito da Ilha Terceira, Serviço de Medicina Interna, Açores, Portugal.; 2 Centro Hospitalar Lisboa Ocidental, Serviço de Infeciologia e Medicina Tropical do Hospital Egas Moniz, Lisboa, Portugal.

A new population of immunocompromised individuals has emerged from increased use of immunosuppressive therapy. The use of corticosteroids associated with other immunosuppressive therapies is a key risk factor for *Pneumocystis jirovecii* pneumonia (PJP) in patients without HIV, and guidelines for treatment and prophylaxis have recently been created. However, cases of PJP in patients undergoing corticosteroid monotherapy are rare^1^.

A 62-year-old man with no relevant personal history was hospitalized for a space-occupying lesion compatible with a brain abscess. A long course of targeted antibiotic therapy was administered and, because of cerebral edema, adjuvant corticosteroids were administered for 6 weeks (cumulative dose of >700 mg).

**FIGURE 1:**
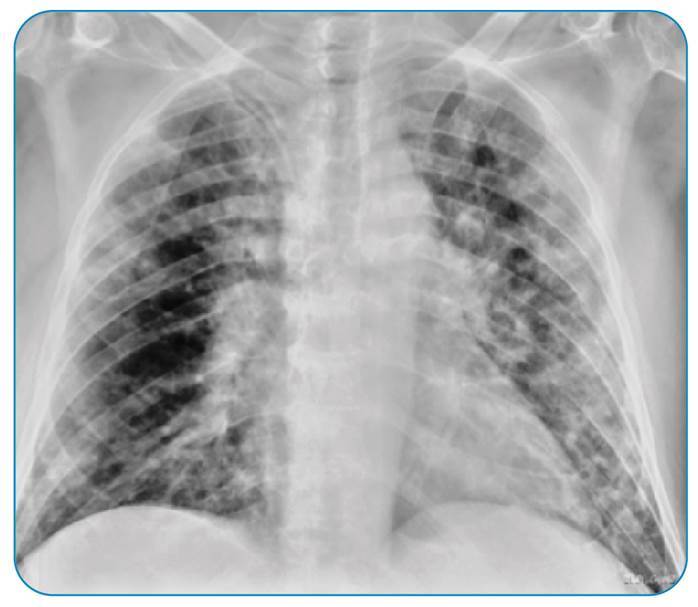
Chest radiograph at admission demonstrating diffuse bilateral interstitial infiltrates.

**FIGURE 2:**
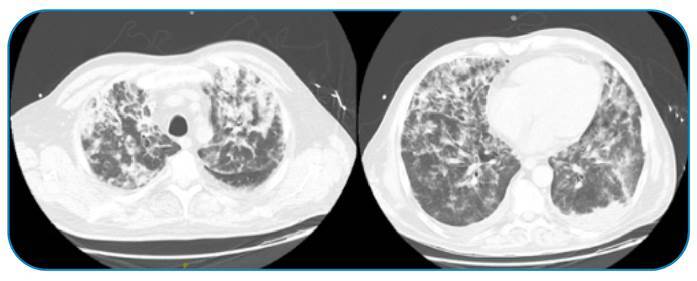
Computed tomography scan from hospital admission demonstrating patchy ground-glass opacities that are more evident in the lower and upper lobes, interspersed with zones of parenchymal consolidation.

Two weeks after discontinuing corticosteroid therapy, the patient presented with fever and respiratory failure. Chest radiography revealed diffuse bilateral interstitial infiltrates (Figure 1), and hospital-acquired pneumonia was diagnosed. Unfortunately, the patient’s clinical status quickly deteriorated; he developed severe respiratory failure, and invasive mechanical ventilation was initiated. Chest computed tomography showed patchy ground-glass opacities, which were more evident in the lower and upper lobes, interspersed with zones of parenchymal consolidation (Figure 2).

Bronchoalveolar lavage was performed, with the identification of *P. jirovecii*.

**FIGURE 3:**
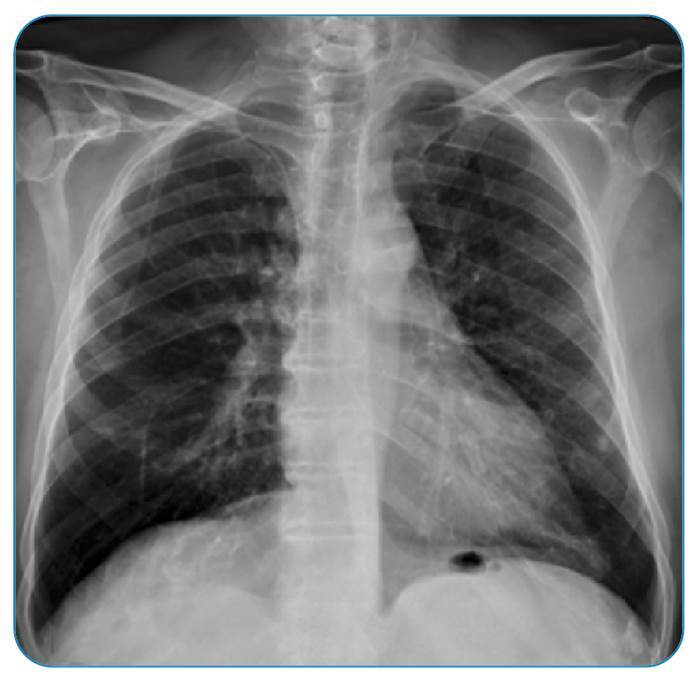
Chest radiograph after 10 days of trimethoprim-sulfamethoxazole treatment.

Ten days after starting targeted therapy, the patient’s clinical status and imaging findings improved (Figure 3).

In patients receiving corticosteroid therapy, the threshold of suspicion of opportunistic infections should be low. Early treatment in this patient prevented clinical deterioration and changed the disease evolution and prognosis^2^.
